# USE OF TRANEXAMIC ACID IN TRAUMA PATIENTS: AN ANALYSIS OF COST-EFFECTIVENESS FOR USE IN BRAZIL

**DOI:** 10.1590/0102-6720201600040017

**Published:** 2016

**Authors:** Marcelo A. PINTO, Jair G. da SILVA, Aljamir D. CHEDID, Marcio F. CHEDID

**Affiliations:** 1 Division of General and Trauma Surgery, Hospital de Pronto Socorro Municipal de Porto Alegre;; 2 Division of Gastrointestinal Surgery and Liver and Pancreas Transplantation, Hospital de Clínicas de Porto Alegre, Federal University of Rio Grande do Sul;; 3 Kidney Transplantation Group, Hospital Santa Casa de Misericórdia de Porto Alegre, Porto Alegre, RS, Brazil

**Keywords:** acid, Trauma, Schock, Operation, Coagulopathy, Cost-benefit analysis.

## Abstract

**Introduction:**

Use of tranexamic acid (TXA) in trauma has been the subject of growing interest by researchers and health professionals. However, there are still several open questions regarding its use. In some aspects medical literature is controversial. The points of disagreement among experts include questions such as: Which patients should receive TXA in trauma? Should treatment be performed in the pre-hospital environment? Is there any need for laboratory parameters before starting TXA treatment? What is the drug safety profile? The main issue on which there is still no basis in literature is: What is the indication for treatment within massive transfusion protocols?

**Objective:**

Answer the questions proposed based on critical evaluation of the evidence gathered so far and carry out a study of cost-effectiveness of TXA use in trauma adapted to the Brazilian reality.

**Methods:**

A literature review was performed through searching Pubmed.com, Embase and Cab Abstract by headings "tranexamic AND trauma", in all languages, yielding 426 articles. Manuscripts reporting on TXA utilization for elective procedures were excluded, remaining 79 articles. Fifty-five articles were selected, and critically evaluated in order to answer study questions. The evaluation of cost effectiveness was performed using CRASH-2 trial data and Brazilian official population data.

**Results:**

TXA is effective and efficient, and should be administered to a wide range of patients, including those with indication evaluated in research protocols and current indication criteria for TXA should be expanded. As for the cost-effectiveness, the TXA proved to be cost-effective with an average cost of R$ 61.35 (currently US$16) per year of life saved.

**Conclusion:**

The use of TXA in trauma setting seems to be effective, efficient and cost-effective in the various groups of polytrauma patients. Its use in massive transfusion protocols should be the subject of further investigations.

## RESUMO

**Introdução:**

O uso do ácido tranexâmico (TXA) no trauma tem sido alvo de interesse crescente por parte de pesquisadores e profissionais de saúde. No entanto, seus benefícios ainda não foram completamente definidos. Os pontos de divergência entre especialistas incluem questões como: quais pacientes devem receber TXA no trauma? O tratamento deve ser realizado em ambiente pré-hospitalar? Há necessidade de exames laboratoriais para indicar o tratamento? Qual o perfil de segurança da droga? A principal questão para a qual ainda não existe qualquer embasamento na literatura é: qual a indicação do tratamento dentro de protocolos de transfusão maciça?

**Objetivo:**

Responder às questões propostas, com base em avaliação crítica da evidência reunida até o momento e realizar estudo de custo-efetividade do uso do TXA no trauma adaptado à realidade brasileira.

**Métodos:**

Foi realizada revisão da literatura através de estratégia de busca: PubMed.com, Embase e no Cab Abstract pelos descritores "tranexamic AND trauma", em todos idiomas, resultando em 426 artigos. Foram excluídos aqueles relativos às operações eletivas, restando 79 artigos. Cinquenta e cinco foram selecionados e avaliados criticamente com vistas a responder às questões em estudo. A avaliação de custo-efetividade foi realizada utilizando dados do estudo CRASH-2 e populacionais oficiais brasileiros.

**Resultados:**

Através da análise da evidência disponível chegou-se à conclusão de que o ácido tranexâmico é tratamento eficaz e efetivo, devendo ser administrado à ampla gama de pacientes, incluindo todos aqueles com indicação já avaliada nos protocolos de pesquisa publicados e provavelmente devam-se expandir os critérios de indicação. Quanto à avaliação de custo-efetividade, o TXA mostrou-se bastante custo-eficaz com gasto médio de R$ 61,35 por ano de vida salvo.

**Conclusão:**

O uso do ácido tranexâmico no trauma parece ser eficaz, efetivo e custo-eficaz nos diversos grupos de pacientes politraumatizados. Seu uso em protocolos de transfusão maciça ainda deve ser objeto de futuras investigações.

## INTRODUCTION

Tranexamic acid (TXA) is a synthetic analogue of the amino acid lysine, discovered in the laboratory in 1962. TXA has low commercial cost, which has favored its routine use for patients presenting with metrorrhagia and for bleeding prevention in patients with hereditary bleeding disorders submitted to dental extraction^2^. More recently, TXA use has been expanded to patients undergoing elective surgery, showing to reduce the needs for blood transfusion^7^.

TXA acts by reversibly binding to lysine receptor sites on plasminogen, thus preventing it to bind to the tissue plasminogen activator (tPA). Once this binding is prevented, plasmin will not be formed, avoiding fibrinolysis^1^.

Randomized controlled trials, cohort studies and meta-analyzes were performed to evaluate and measure the potential effects of TXA utilization in trauma patients^3^ ^,^ ^5^ ^,^ ^20^ ^,^ ^23^.

Because of the universal distribution of trauma and its concentration in low and middle per capita income countries, it becomes important to evaluate cost-effectiveness of TXA in this scenario. Studies evaluating cost-effectiveness of TXA in different Countries have been published. However there are no prior studies evaluating the Brazilian reality with its peculiarities that hinder its comparison to other countries of lower income per capita^6^.

This study aimed to review the accumulated knowledge in the use of TXA and respond to issues that are relevant to clinical practice. In addition it is intended to make an analysis of the cost effectiveness of TXA use specifically to Brazilian reality.

## METHODS

Search strategy: PubMed, Medline, Embase and Cab Abstract by the terms "tranexamic AND trauma" in all languages, resulting in 426 articles. Those related to elective surgery were excluded, remaining 79 articles. After excluding those with irrelevant content for the purpose of this study, 55 articles have remained. Among these, there were seven original articles reporting on intervention studies, two Cochrane meta-analysis, an one cost-effectiveness study, six review articles and six subgroup analyzes from previous studies.

Evaluation of cost effectiveness of TXA was performed by calculating the incremental cost of treatment divided by the number of years of life gained with the intervention. The determination of the incremental cost of treatment, i.e., the extra amount to be spent on the implementation of the treatment compared to standard treatment (without use of TXA) was performed ​​taking into account the estimated costs for the treatment of patients with TXA in the dose recommended by the study "Effects of tranexamic acid in death", vascular occlusive events, and blood transfusion in trauma Patients with significant hemorrhage (CRASH-2)^5^, i.e. 1 g EV dose plus 1 g IV after eight hours. The spent considered standard treatment for application of TXA was eight 250mg vials of TXA, two 40X12 mm needles, two 20 ml syringes, one regular infusion set, one micro i.e. infusion set and two 100 ml bottles of saline. Unlike what was done in previous studies incremental spending on labor on TXA infusion was not considered because the Brazilian health public system (SUS), as it is organized, allows small procedures to be added to the care practice without requiring to hire new staff. To determine the incremental cost of treatment, budget was performed by utilizing TXA vials obtained from leading manufacturers and laboratories. Other materials needed for administration of TXA had their values ​​determined by consulting the lower values ​​of online auction. To determine the average incremental cost of treatment simple arithmetic average of the highest and lowest value found was performed.

As for calculating the number of years of life gained with the treatment the following equation was utilized:

Number of years of life gained = (average Brazilian life expectancy - mean age of the CRASH-2 patients) / (NNT from CRASH-2)

In this equation, NNT is the number of patients to be treated to prevent one death in CRASH-2 study. In other words, NNT is the inverse of the absolute risk reduction. Having the number of life years gained and average incremental cost of treatment, we can directly estimate the cost-effectiveness using the formula below:

Cost-effectiveness = (average price of treatment) / number of years life gained)

in which the cost-effectiveness is given in R$ (Brazilian currency) per life year gained.

This formula was utilized instead of a parametric function Gompertz used in other cost-effectiveness study published on the subject^6^ because of its simplicity, for disregard of computing resources for its calculation and because it fits well to reality. In addition to that, almost all of the deaths related to trauma occurring in the CRASH-2 study follow-up period and that the group of patients included in this study should not differ statistically with respect to age of patients seen in Brazilian trauma centers. The NNT data comes from the CRASH-2 study, which are the most reliable. To determine the expected average Brazilian life, used in conjunction with the NNT for calculating the number of years of life gained with treatment, 2014 official data were used.

## RESULTS

### Literature review

According to meta-analysis there is no statistically significant relationship between use of TXA and mortality reduction in elective surgeries. However, the low prevalence of death secondary to bleeding in elective surgeries may have contributed to make the magnitude of a possible benefit not possible to estimate due to the statistical power of the studies^3^. However, this same review showed benefits in outcomes such as need for blood transfusion (reduced by one third), volume of blood transfusion (reduced by one pack or red cells concentrate per patient) and need for surgical reoperation due to bleeding (halved)^3^. In polytrauma victims, due to unscheduled nature of tissue damage, there is a greater tendency for bleeding secondary to small vessel trauma if compared to the controlled damage generated by elective surgery. Thus, it is likely that, as has been proven in elective surgeries, there may be benefit in the administration of tranexamic acid. Additionally, since the death outcome secondary to bleeding is much more common in trauma patients than in patients undergoing elective surgical procedures, it was even possible to assume that the benefit not observed mortality in the meta-analysis for elective surgery would be obtained for trauma patients.

An additional meta-analysis in the context of antifibrinolytic use in trauma was conducted^20^. The evidence gathered at the time was insufficient to prove the effectiveness of antifibrinolytic use due to the small amount of existing studies and the small number of patients evaluated by them. In 2010, a large randomized clinical trial (RCT), double-blind, placebo-controlled, the CRASH-2 study evaluated 20,211 trauma victims. This study was able to demonstrate the benefits of the use of TXA on overall mortality and also for mortality related to bleeding in the trauma setting. It was thus confirmed the theoretical benefit proposed above. However, surprisingly, this study showed no decrease in the need or the amount of blood products for the care of patients^5^. After this study was published, a new meta-analysis was performed^20^. After excluding inappropriate studies or studies with unacceptable biases and also by adding patients of CRASH-2 this new meta-analysis revealed different conclusions than those of from the 2008 review^20^. Due to the huge difference in sample space between the two studies eligible for this review (77 against 20211) the data produced resulted almost identical to the CRASH-2^5^ ^,^ ^20^. Soon after, a retrospective observational study evaluating the use of TXA in military environment, the military Application of Tranexamic Acid in Trauma Emergency Resuscitation (MATTERs, 2012) was published^14^. Subgroup analysis of this study compared use of TXA and use of TXA associated with cryoprecipitate and showed encouraging results, suggesting additional benefit with combined use of cryoprecipitate and TXA^15^. However, because it is not an ECR and only included patients with serious injuries in military environment, such data are not subject to comparison with those generated by the aforementioned studies. There are no randomized trials evaluating the use of TXA in the trauma setting.

There remain many questions to be answered about the use of TXA in the trauma setting. No information is available on the efficacy and effectiveness of the association of TXA and blood products or its use in massive transfusion protocols. There is no evidence from controlled studies of the possible synergistic effect observed between TXA and cryoprecipitate evaluated by the MATTERs study; there is no data on the use of TXA in patients undergoing damage control surgery*.* Furthermore, there are no specific data specifically evaluating subgroups of patients suffering from penetrating or blunt injury. The mechanism by which this drug acts in trauma, especially with regard to its anti-inflammatory effect remains uncertain^2^. Also, the efficiency and effectiveness of TXA as a drug for prehospital use has not been established yet.

### Key issues

#### Mortality prevention: When should TXA be indicated? 

The largest benefit in mortality prevention with the use of TXA is observed in patients with established coagulopathy and severe trauma^5^ ^,^ ^8^ ^,^ ^12^. This prompted researchers to question whether indication of TXA should or not be restricted in this subgroup of patients. The authors of this study agree with Roberts^19^ ^,^ ^21^ when he advocates the use of TXA in all trauma patients with bleeding or risk of bleeding^18^. Since differences in systolic blood pressure of patients does not generate statistical difference in mortality secondary to hemorrhage (main mechanism of action of treatment, p=0.33)^21^ remains clear that this is effective in all degrees of shock, i.e., their biological effect is exerted almost uniformly in all patients regardless of their systolic pressure. Thus, the difference in mortality from all causes identified among the sickest patients seems to be a mere statistical artifact secondary to higher death rate in the group of critically ill patients, it seems logical to indicate treatment in all groups of patients suffering shock, since this can prevent the less sick patients to evolve to higher degrees of shock, like III or IV and, only then, be consider eligible for the use of TXA. This strategy seems more appropriate since there is a clear relationship between early use of TXA and better clinical outcomes^5^.

#### Potential increase in the risk of thromboembolic events 

Although the CRASH-2 study did not report statistically significant differences with regards to thromboembolic events in the intervention and control^5^ groups, there is biological plausibility for an increase in thromboembolic events in patients being treated with TXA. As occurred with the use of aprotinin, a drug structurally similar to TXA, increased risk of thromboembolic events may appear in later studies^13^. The results of the MATTERs study showed increased occurrence of these events for patients receiving TXA vs. controls: 0.3% of pulmonary thromboembolism in the control group vs. 2.7% (p=0.01) in TXA group, and 0.2% of deep venous thrombosis in the TXA group vs. 2.4% (p=0.01) in controls for deep venous thrombosis^15^. There are two key issues to comment on disparity of the results of between these two studies. As for CRASH-2 data, since the most 274 centers involved in the study were located in low- and middle - income per capita^5^ and thus with less access to diagnostic methods, it is possible to have occurred under-reporting of events. This potentially could have masked a possible increased risk of thromboembolic events. Moreover, patients enrolled in MATTERs were not randomized, some patients being relocated to intervention or to placebo groups^14^ during the study. There was clearly a sicker group of patients who received TXA compared to the group that received no intervention. It may be seen by comparing the number of patients with Glasgow Coma Score ≤8, equal to 63.3% in the intervention group versus 35.6% in the control group (p<0.001), the ISS (injury severity score) average with 25.2 versus 22.5 (p<0.001)^15^, the RTS (revised trauma score) 5.53 to 6.04 (p=0.01), and the number of patients with systolic blood pressure less than 90 mmHg, 22.8% vs. 13.8% (p=0.003)^15^. The group receiving TXA was sicker, as widely documented. Although it makes even more robust the reduction in mortality obtained by the use of TXA, it casts doubt on the increase in thromboembolic events observed in the treatment group. This increase could be due to potential effect of TXA or could simply derive from the greater severity of the patients in this group or even could derive from the combination of these two causes. Reinforcing this point of view, adjusting the risk of patients by severity, the thromboembolic risk ratio potentially could turn to not be statistically significant^18^. Analyzing the two newly exposed factors, it is clear that both the evidence of the CRASH-2, contrary to the existence of a prothrombotic effect^5^, and the evidence of MATTERs favorable to the existence of this effect may not seem reliable^15^.

Only one study was designed with the main objective to assess thromboembolic events in trauma patients treated with TXA. This is an observational study that evaluated 115 patients at high risk for such events. This study found no difference in the occurrence of thromboembolic events among patients receiving TXA vs. those not receiving the drug (p=0.788)^25^. Although it comes from an observational study, it apparently presents with appropriate design and brings reliable data to determine the safety of the drug. Finally, we should remember that the CRASH-2 study showed small but statistically significant reduction in the incidence of acute myocardial infarction in the treatment group^5^. This benefit may be due to lower oxygen consumption by the myocardium in patients whose bleeding was reduced by the action of the drug^9^. Thus, the accumulated evidence to date points to the safety of treatment with TXA; however, there is still need for further RCTs designed to evaluate the occurrence of thromboembolic events as the primary outcome and the potential reduction in the occurrence of myocardial infarction or other ischemic events.

#### Criteria for therapy with TXA use 

Both the CRASH-2 study and the MATTERs study utilized clinical parameters to indicate the treatment or inclusion in the study^5^ ^,^ ^14^. Criticism was directed to the authors of the former study, pointing out that laboratory parameters should better evaluate patients with hyperfibrinolysis and thus they should be better indicators of which patients would benefit most from the treatment^17^. The authors of the present study disagree with this point of view, since the decision making in trauma cannot always expect results of laboratory tests, which are not always readily available. It is considered one of the strengths of the CRASH-2, the clinical scenario in which it was done. Regarding its multicentric nature and the prevalence of low and middle per capita income countries among the participants, the study accurately portraits the reality where victims of trauma are concentrated around the world. The disadvantage of including patients who are not at hyperfibrinolysis state in the study is to reduce the magnitude of benefit. If, even with this possible reduction of the magnitude of this benefit was even gaugeable and significant, the greater should be the benefit brought to patients for treatment. Furthermore, there is no diagnostic test specific and 100% sensitive. Thus, expecting the results from laboratory tests to start TXA therapy, could deny potentially life-saving therapeutic interventions to patients who could benefits from these. On the other hand, when a treatment of low cost and high safety profile as TXA is overprescribed, the risks are is likely to be outweighed by the benefits. Even though the potential benefits of thromboelastography and thromboelastometry cannot be denied^17^, only it is argued here that the data from these methods will find their best application for the indication of high cost or low therapeutic index treatments such as the use of blood derivatives. In summary, the use of clinical parameters for inclusion of patients in the study, namely the use of clinical criteria for treatment indication, criticized by some researchers, seem least one study design defect than an additional factor that increases the external validity of the study.

#### Protocols of massive transfusion 

There are no studies directly evaluating the use of TXA in massive transfusion protocols. The only data available related to the subject comes from subgroup analysis of the MATTERs study^15^. In the study, the use of TXA in combination with cryoprecipitate showed a reduction in mortality when compared to the individual use of each and to the control group. It was also demonstrated an independent effect for each intervention. Data obtained through logistic regression showed that the odds ratio towards death for the treatment group receiving isolate cryoprecipitate as compared to the absence of treatment was 0.61 (p=0.02), being 0.61 (p=0.01) for TXA and 0.38 (p<0.01) for the combination of both^15^. This profile raised the possibility of synergism between cryoprecipitate and TXA, which was tested by the same researchers, without obtaining the statistical significance achieved in this assessment of synergism (p=0,21). Although the existence of interaction was not proved, it cannot be rejected, raising the need for further studies in this subject.

#### Prehospital Care 

A recent study in the prehospital care with medical evacuation by helicopter was published in 2013^26^. Inclusion criteria for this study were: patients older than 16 years of age, heart rate of more than 110 beats per minute and systolic blood pressure of less than 90 mmHg (very similar criteria to the ones from CRASH-2 study)^5^ ^,^ ^26^. The intervention included TXA administration to all patients during transport, associated to treatment with permissive hypotension in those with injuries in the chest and abdomen. This study evaluated 13 patients who were administered TXA in an average time of 32 min after the arrival of the team to the scene. As the mean time to response was reported as 33 min, it is concluded that TXA was administered on average 65 min after injury. The authors did not report any adverse effects related to intervention^26^.

A cohort reporting the use of TXA in the prehospital environment by Israeli defense forces also was published^11^. Forty cases were reported, in whom treatment was started as soon as possible without delay^11^. Inclusion criteria were: penetrating wound in the neck, chest, abdomen or pelvis or any penetrating or blunt injury accompanied by signs of shock. The authors defined shock as systolic pressure of less than 90 mmHg, heart rate above 100 beats per minute in two consecutive measurements, peripheral capillary refill greater than 2 s or altered level of consciousness not associated with blunt TBI^11^. Clearly the authors used much broader inclusion criteria than those adopted by the CRASH-2^5^ ^,^ ^11^. There were no reports of adverse effects related to treatment, which increases the importance of the findings, since there were liberal criteria to indicate treatment in this study^11^. The authors emphasized the importance of the safety profile of TXA, since in their point of view, prehospital care teams tend to overprescribe drugs^11^.

From the data of both studies, which together totalized 53 patients, it can be concluded that, although the published experience is still modest, it seems safe to use TXA in the prehospital environment. Note also that the average use time of the trauma was 65 min in the first study (no data concerning this second study), that is, the upper limit of subgroup analysis showed greater benefit in the CRASH-2. Similarly, recently published study reports on a series of 20 consecutive patients receiving TXA during prehospital aeromedical transports with no adverse effects^16^. Thus, due to the apparent lack of serious adverse effects and the superiority of the treatment provided in the first hour^5^, it seems that the prehospital environment would be an appropriate setting for the use of TXA in trauma. With the publication of PATCH study more data should be available, helping to dissolve the still lingering doubts concerning the use of TXA in prehospital care.

### Cost-effectiveness analysis of the use of TXA

The potential of avoiding a large number of deaths using the TXA has been identified by various researchers. Estimates based on CRASH-2 data and epidemiological information from the World Health Organization (WHO) come to point out the possibility of avoiding 128,000 of the 400,000 annual deaths that occur in bleeding trauma patients around the world^22^. Study evaluating the cost-effectiveness of the use of TXA in trauma, in three different scenarios, was conducted: England (high gross domestic product, GDP, per capita), India (middle GDP per capita) and Tanzania (low GDP per capita)^6^. However it does not seem possible to extrapolate these results to the Brazilian reality.

In order to clarify some of these questions, this study aims to make a cost-effectiveness evaluation of TXA use in trauma in Brazil.

To evaluate the price of TXA, a survey of prices of TXA provided by different laboratories, Pfizer (reference product) Nikkho (similar), Hipolabor (generic) and EMS (generic) was conducted. Evaluation of official data published online trading days from January 2013 to December 2014, for vials of 250 mg of TXA. Costs related to the other materials required for the administration of TXA described above were estimated through evaluating the results of official electronic auctions published between January 2013 and December 2014. For the study purposes only winning proposals for each one of the sessions were considered, as they represent the actual value paid in acquisitions made ​​by Brazilian public hospitals responsible for the care of most trauma patients in Brazilian public healthcare system. The calculated values ​​are shown in Table 1.

**TABLE 1:** Incremental cost of TXA treatment

Product	Necessary amount	Biggest price	Lowest price	Higher total price	Lowest total price
Tranexamic acid 250mg/5 ml	8 vials	R$ 6.00	R$ 1.97	R$ 48.00	R$ 15.76
Needle 40X12 mm, or similar	2 units	R$ 0.14	R$ 0.08	R$ 0.28	R$ 0.16
Syringe 20 ml	2 units	R$ 0.49	R$ 0.22	R$ 0.98	R$ 0.44
100 ml physiological saline	2 units	R$ 1.36	R$ 0.89	R$ 2.72	R$ 1.78
Regular infusion set	1 unit	R$ 1.17	R$ 0.67	R$ 1.17	R$ 0.67
Micro i.v. infusion set	1 unit	R$ 17.00	R$ 14.06	R$ 17.00	R$ 14.06
Total expenditure	-	-	-	R$ 70.15	R$ 32.87

The 2014 official data estimates the Brazilian life expectancy at 74.9 years. Using the data above, the information of the CRASH-2 study and the formula presented in the section above, the following value were obtained: R$ 54.65 per life year gained using the lower available prices, and R$ 116.63 using If the higher available prices resulting in an average of R$ 85.64. It is still possible to infer that the incremental cost of treatment with TXA to save a life ranges from R$ 2,202.29 to R$ 4,700.05, with an average of R$ 3,451.17. For comparison purposes, a study calculated the cost per year of life gained for $ 48 (international dollars) in Tanzania, $ 66 in India and $ 64 in the United Kingdom^6^. It may be highlighted that the data are from 2011 and the researchers used even older TXA quotations, with prices ranging between $ 2.57 and $ 45.67 for drug 2 g, very different from R$ 15.76 to R$ 48.00 practiced in Brazil.

## CONCLUSION

The effectiveness of TXA in trauma seems well established (level of evidence A)^5^ ^,^ ^20^. The effect of the drug seems to extend to patients in military environment (level of evidence C)^14^. Although there are no studies that discriminate patients suffering from penetrating and blunt trauma, there seems to be an indication of the use of TXA in both groups (level of evidence A)^5^ ^,^ ^20^. Thus, the need for studies separating these two groups (penetrating vs. blunt trauma patients) is more important for epidemiological purposes than for driving clinical practice. There is evidence of the safety of the use of tranexamic acid in the prehospital setting (level of evidence C)^11^ ^,^ ^26^. The authors of this study believe that, since that there is enough evidence that the sooner TXA is used, the greater the benefit it provides, additional studies comparing the effectiveness of the drug administered in the prehospital environment with its use inside the hospital are not necessary^5^ ^,^ ^24^. However, for TXA inclusion in prehospital care protocols, new studies evaluating the safety of treatment would be warranted, since the available studies have included a small number of patients.

This study concluded that the use of TXA is highly cost-effective, and the average cost for each year of life saved is estimated at R$ 61.35. This data highly justifies the adoption of TXA treatment for polytrauma protocols, following the prescription model used by the CRASH-2 teams, and entails conducting studies to assess the cost-effectiveness of protocols with even more comprehensive indication criteria for this drug.

## References

[1] Boström J, Grant JA, Fjellström O, Thelin A, Gustafsson D (2013). Potent fibrinolysis inhibitor discovered by shape and electrostatic complementarity to the drug tranexamic acid. J Med Chem.

[2] Cap AP, Baer DG, Orman JA, Aden J, Ryan K, Blackbourne LH (2011). Tranexamic acid for trauma patients a critical review of the literature. J Trauma.

[3] Coats T, Roberts IG (2011). Antifibrinolytic drugs for acute traumatic injury (Review).. The Cochrane Collaboration.

[4] Roberts I, Shakur H, Afolabi A, Brohi K, Coats T, Dewan Y, Gando S, Guyatt G, Hunt BJ, Morales C, Perel P, Prieto-Merino D, Woolley T., CRASH-2 collaborators (2011). The importance of early treatment with tranexamic acid in bleeding trauma patients: an exploratory analysis of the CRASH-2 randomised controlled trial. Lancet.

[5] Shakur H, Roberts I, Bautista R, Caballero J, Coats T, Dewan Y, El-Sayed H, Gogichaishvili T, Gupta S, Herrera J, Hunt B, Iribhogbe P, Izurieta M, Khamis H, Komolafe E, Marrero MA, Mejía-Mantilla J, Miranda J, Morales C, Olaomi O, Olldashi F, Perel P, Peto R, Ramana PV, Ravi RR, Yutthakasemsunt S, CRASH-2 trial collaborators (2010). Effects of tranexamic acid on death, vascular occlusive events, and blood transfusion in trauma patients with significant haemorrhage (CRASH-2) a randomised, placebo-controlled trial. Lancet.

[6] Guerriero C, Cairns J, Perel P, Shakur H, Roberts I, CRASH 2 trial collaborators (2011). Cost-effectiveness analysis of administering tranexamic acid to bleeding trauma patients using evidence from the CRASH-2 trial. PLoS One.

[7] Henry DA, Carless PA, Moxey AJ, O'Connell D, Stokes BJ, McClelland B, Laupacis A, Fergusson D (2007). Anti-fibrinolytic use for minimising perioperative allogeneic blood transfusion. Cochrane Database Syst Rev.

[8] Harvey V, Perrone J, Kim P (2014). Does the use of tranexamic acid improve trauma mortality. Ann Emerg Med.

[9] Hunt BJ (2015). The current place of tranexamic acid in the management of bleeding. Anaesthesia.

[10] Ker K, Kiriya J, Perel P, Edwards P, Shakur H, Roberts I (2012). Avoidable mortality from giving tranexamic acid to bleeding trauma patients an estimation based on WHO mortality data, a systematic literature review and data from the CRASH-2 trial. BMC Emerg Med.

[11] Lipsky AM, Abramovich A, Nadler R, Feinstein U, Shaked G, Kreiss Y, Glassberg E (2014). Tranexamic acid in the prehospital setting Israel Defense Forces' initial experience. Injury.

[12] Mejia-Mantilla JH, Puentes-Manosalva FE, Ciro JD, Morales C (2009). Hemorragia y trauma, avances del estudio CRASH-2 en Colombia. Rev Colomb de Cirurgia.

[13] Mitra B, Mazur S, Cameron PA, Bernard S, Burns B, Smith A, Rashford S, Fitzgerald M, Smith K, Gruen RL, PATCH-Trauma Study Investigators (2014). Tranexamic acid for trauma filling the 'GAP' in evidence. Emerg Med Australas.

[14] Morrison JJ, Dubose JJ, Rasmussen TE, Midwinter MJ (2012). Military Application of Tranexamic Acid in Trauma Emergency Resuscitation (MATTERs) Study. Arch Surg.

[15] Morrison JJ, Ross JD, Dubose JJ, Jansen JO, Midwinter MJ, Rasmussen TE (2013). Association of cryoprecipitate and tranexamic acid with improved survival following wartime injury findings from the MATTERs II Study. JAMA Surg.

[16] Mrochuk M, ÓDochartaigh D, Chang E (2015). Rural trauma patients cannot wait tranexamic Acid administration by helicopter emergency medical services. Air Med J.

[17] Napolitano LM, Cohen MJ, Cotton BA, Schreiber MA, Moore EE (2013). Tranexamic acid in trauma how should we use it?. J Trauma Acute Care Surg.

[18] Roberts Ian (2012). The case for universal access to tranexamic acid. International Society of Blood Transfusion, ISBT Science Series.

[19] Roberts I, Perel P, Prieto-Merino D, Shakur H, Coats T, Hunt BJ, Lecky F, Brohi K, Willett K, CRASH-2 Collaborators (2012). Effect of tranexamic acid on mortality in patients with traumatic bleeding prespecified analysis of data from randomised controlled trial. BMJ.

[20] Roberts I, Shakur H, Ker K, Coats T, CRASH-2 Trial collaborators (2012). Antifibrinolytic drugs for acute traumatic injury. Cochrane Database Syst Rev.

[21] Roberts I, Prieto-Merino D (2014t). Applying results from clinical trials: tranexamic acid in trauma patients. J Intensive Care.

[22] Roberts I, Prieto-Merino D, Manno D (2014). Mechanism of action of tranexamic acid in bleeding trauma patients an exploratory analysis of data from the CRASH-2 trial. Crit Care.

[23] Sobral Felipe Antonio (2016). Tranexamic acid action on liver regeneration after partial hepatectomy: experimental model in rats. Arq Bras Cir Dig.

[24] Valle EJ, Allen CJ, Van Haren RM, Jouria JM, Li H, Livingstone AS, Namias N, Schulman CI, Proctor KG (2014). Do all trauma patients benefit from tranexamic acid. J Trauma Acute Care Surg.

[25] Van Haren RM, Valle E, Busko AM, Guarch GA, Jouria JA, Namias N, Livingstone AS, Proctor KG (2013). Safety and efficacy of tranexamic acid in trauma patients at high risk for venous thromboembolism?.

[26] Vu EN, Schlamp RS, Wand RT, Kleine-Deters GA, Vu MP, Tallon JM (2013). Prehospital use of tranexamic acid for hemorrhagic shock in primary and secondary air medical evacuation. Air Med J.

